# Battling ingroup bias with effective intergroup leadership

**DOI:** 10.1111/bjso.12445

**Published:** 2021-01-20

**Authors:** Christine Kershaw, David E. Rast, Michael A. Hogg, Daan van Knippenberg

**Affiliations:** ^1^ University of Alberta Edmonton Alberta Canada; ^2^ Claremont Graduate University Claremont California USA; ^3^ Drexel University Philadelphia Pennsylvania USA

**Keywords:** intergroup relations, social identity, intergroup leadership theory, intergroup relational identity

## Abstract

Intergroup conflict and bias often occur between subgroups nested within a superordinate group. In these situations, the leader of the superordinate group plays a key role, as an intergroup leader, in reducing conflict. To be effective, an intergroup leader should avoid (1) threatening the subgroups' distinctive identities, and (2) being viewed by one or both groups as ‘one of them’ rather than ‘one of us’. Intergroup leadership theory (*Acad Manag Rev*, **37**, 2012a, 232) posits intergroup leaders can improve subgroup relations by promoting an *intergroup relational identity*. Two studies (*N*s = 178 and 223) tested whether an out‐subgroup or in‐subgroup leader could improve intergroup attitudes, even among strong subgroup identifiers, by promoting either an intergroup relational identity or a collective identity. We hypothesized an interaction of these variables demonstrating the effectiveness of an intergroup relational identity message for an out‐subgroup leader in lessening ingroup bias, especially among strong subgroup identification. Our results, and a meta‐analytic summary across both studies (*N* = 401), supported our hypothesis and intergroup leadership theory, demonstrating an intergroup relational identity is an effective strategy for improving intergroup relations.

## Background

Social groups, whether large or small, tend to be structured into distinct subgroups. Subgroup identities are configured and impacted by their shared superordinate group membership and identity. This is true for at the national and international level; for example, the European Union embraces 28 separate member states, the nation of Sri Lanka embraces Tamil and Singhalese ethnic subgroups, and the US embraces Democrats, Republicans, and a number of other smaller political entities. It is also true for organizations both at the inter‐organizational level such as in joint ventures and alliances, and at the intra‐organizational level where different units such as departments and divisions and different groups such as doctors and nurses in a hospital are expected to jointly produce outcomes from distinct group roles. As a result, intergroup relations are often inter‐subgroup relations because these relations are between groups that are part of the same overarching superordinate group. Ideally, that shared superordinate group membership would ensure that inter‐subgroup relations would be smooth; in practice, what we observe despite the shared superordinate group membership is that intergroup relations are challenging and hampered by, in which people tend to be biased in favour of their own group. This bias can be relatively innocuous, but it can also run deep and be associated with polarized identities, deep outgroup antipathy, and destructive actions (Dovidio & Gaertner, 2010; van Knippenberg, 2003; van Knippenberg & Hogg, 2018; Yzerbyt & Demoulin, 2010).

An intergroup leader, whether they are facing political parties, organizational groups, or other subgroups, is challenged to address inter‐subgroup relations. The question investigated in this article is the role played by the superordinate group's leader (e.g., national leader, organizational leader) in exacerbating or ameliorating destructive inter‐subgroup relations, such as those occurring between different ethnic groups or organizational divisions. A particular challenge intergroup leaders typically face is that they are more strongly associated with one subgroup relative to other subgroups. For example, the organizational leader has a background in one of the departments, or the national leader is affiliated with one of the political parties or ethnic groups. An intergroup leader's effectiveness will be influenced by their subgroup affiliation, especially as subgroup members more strongly identify with their subgroups (Hogg, van Knippenberg, & Rast, 2012b). In particular, we explore (1) the subgroup affiliation of the leader, (2) what superordinate identity message the leader delivers (a core element of intergroup leadership identified in intergroup leadership theory; Hogg, van Knippenberg, & Rast, 2012a), and (3) how strongly identified people are with their subgroups.

We report two studies that explore the interactive effect of these variables on intergroup attitudes and test predictions derived from a relatively recent theory of intergroup leadership (Hogg, 2015; Hogg et al., 2012a) by focusing on the challenges an out‐subgroup faces in an intergroup leadership context. Currently, testing of this theory has focused on an intergroup leader in which the leader happens to be the in‐subgroup leader for the participants (e.g., Rast, Hogg, & van Knippenberg, 2018). Intergroup leaders, however, are an out‐subgroup leader to at least one of the subgroups to which the participants do not belong. Understanding the influence and limitations of an out‐subgroup intergroup leader – that is, a context where a leader is clearly an in‐ or out‐subgroup leader to the participants – is crucial to intergroup leadership theory. Subgroup identification may also play a key role in intergroup leadership because strong identifiers, relative to weak identifiers, are more likely to derogate out‐subgroup members (e.g., Jetten, Spears, & Manstead, 1997), which would make effective intergroup leadership more challenging. The two studies differ in the intergroup context and methodology employed, addressing the contemporary call for replicability in social psychology (e.g., Open Science Collaboration, 2015; Stangor & Lemay, 2016).

### Improving intergroup relations

Social psychology has long sought to understand the psychology of prejudice and intergroup conflict to reduce prejudice and improve intergroup relations (e.g., Dovidio & Gaertner, 2010; Yzerbyt & Demoulin, 2010). One way to improve intergroup attitudes and relations is by reducing or modifying intergroup bias. Intergroup bias can be considered in terms of evaluation, such as prejudice, behaviour, either explicit or implicit, and valence, either positive (e.g., ingroup favouritism) or negative (e.g., outgroup derogation; Dovidio & Gaertner, 2010). This bias is particularly apparent when group members strongly identify with their group (e.g., Jetten et al., 1997). The challenge is that the very existence of ingroups and outgroups seems to automatically predispose people to be both evaluatively and behaviourally biased in favour of their own group over outgroups.

Social categorization *per se* has repeatedly been shown to generate evaluative and behavioural ingroup favouritism (e.g., Mullen, Brown, & Smith, 1992; Tajfel, 1970; also see Abrams & Hogg, 2010; Kerr, Ao, Hogg, & Zhang, 2018), and the effect seems to occur automatically (Otten & Wentura, 1999). The history and nature of intergroup relations, however, can influence how bias is expressed (e.g., Ellemers, 1993; Tajfel & Turner, 1986). In particular, when relations between groups are viewed as highly competitive (effectively zero‐sum) and hostile, in‐subgroup favouritism tends to be increasingly expressed as extreme evaluative and behavioural discrimination against the out‐subgroup (Mummendey & Otten, 1998).

Given the psychologically deep‐rooted nature of competitive and often hostile intergroup relations, it can be difficult to disarm stereotypes and reduce bias, favouritism, and discrimination. Nevertheless, an enormous amount of social psychological research has focused, with some success, on improving intergroup relations. For example, according to the common ingroup identity model, if one can transform an intergroup context defined by conflicting identities into an intragroup context defined by shared social identity then hostile intergroup relations become harmonious intragroup relations (e.g., Gaertner & Dovidio, 2000; Riek, Mania, Gaertner, McDonald, & Lamoreaux, 2010). Successfully constructing and sustaining a common ingroup identity out of warring factions, however, is not easy. People often protect the distinctiveness of their cherished social identities and strongly resist attempts, which are viewed as a threat, to merge them (see Hornsey & Hogg, 2000a, 2000b); conflict that can be driven by minority groups' attempts to maintain their identities (Bilali, 2014; Verkuyen & Martinovic, 2016) or by strong identifiers' need for distinctiveness (Jetten & Spears, 2003). Other research on improving intergroup relations focuses on intergroup contact: the palliative power of interacting and developing positive interpersonal relations with out‐subgroup members. If the right boundary conditions are met, contact can improve relations (Brown & Hewstone, 2005; Crisp & Abrams, 2008; Dovidio, Eller, & Hewstone, 2011; Pettigrew & Tropp, 2006; Turner & Cameron, 2016), and merely observing or even just imagining successful contact can be effective (Crisp & Turner, 2012; Miles & Crisp, 2014; Zhou, Page‐Gould, Aron, Mayer, & Hewstone, 2019). The problem with this perspective is that the very processes leading to intergroup biases also invite resistance to establishing these favourable contact conditions (e.g., the desire to achieve and maintain a higher‐status position for the own group; Ellemers, 1993). Moreover, a collective identity may often be subject to ingroup projection mechanisms (Mummendey & Wenzel, 1999; Wenzel, Mummendey, & Walszus, 2007): to the extent that there is a dominant (i.e., higher status, more powerful) subgroup, this subgroup may stake claims about the collective identity that, in essence, suggests that the collective identity is largely defined by the dominant subgroup’s identity at the expense of intergroup relations (e.g., the rise of Donald Trump to the US Presidency has led commentators to reflect on how to his largely White base American identity seems defined by the identity of White Americans as a subgroup, which carries the implications that Whites are ‘more American’ than other ethnic groups and as a result does not invite better relationships between different ethnic groups in the US).

### A role for intergroup leadership

Intergroup relations concepts and theories typically ignore the role of leadership, yet a leader is a ubiquitous feature of group and intergroup phenomena. Practically, all groups have some form of leadership and people look to their leaders to define and communicate their group's identity (e.g., Hogg, 2018a). Of particular relevance, and the focus of this article, is the role played by the leader of a superordinate group comprised of two or more distinct subgroups (i.e., an intergroup leader) in reducing inter‐subgroup conflict. One way a leader can influence intergroup relations is through identity‐relevant information provided in a leader's rhetoric, rhetoric delineating and articulating the group's identity.

Unfortunately, exploring the role of leaders in improving or worsening intergroup relations is largely missing (cf. Pittinsky, 2009). Leaders are particularly important because of the influence they have within a group and their group's relation with other groups. When group membership is particularly important or salient, group members look to their leaders to see how they ought to behave (e.g., Hogg, 2015; Hogg et al., 2012b). Although theories provide pathways to improving intergroup relations, such as promoting/creating different types of social identities, reframing/recategorizing the intergroup context, developing cross‐group friendships or having real or imagined contact with out‐subgroup members, none of them discuss how to achieve these or who leads the group towards these activities. Outside of these theories, society acknowledges the role leaders play at improving ingroup relations. For instance, leaders such as MLK Jr. and Ghandi are immortalized and celebrated for their leadership bridging the divide between conflict‐ridden groups. Leaders, however, do not only have a positive impact on intergroup relations, they can and do often worsen intergroup relations. For instance, it is well‐documented that US President Donald Trump engaged in behaviours and rhetoric widening the divide between Democrats and Republicans (e.g., Goethals, 2018; Pettigrew, 2017). Unlike traditional intergroup relations theories, intergroup leadership theory (Hogg et al., 2012a) places leaders front and centre in engaging in behaviours and promoting rhetoric to shape inter‐subgroup interactions and engagement. Intergroup leadership theory draws on the social identity perspective that lies at the core of the analysis of intergroup relations and complements the social identity theory of leadership (Hogg et al.., 2012b). The latter focuses on intragroup leadership while the former focuses on intergroup leadership. Intergroup leadership theory proposes that fostering inter‐subgroup relations comes with different challenges than fostering intragroup relationships and therefore different leadership is required to set the stage for effective intergroup collaboration than for effective intragroup efforts.

Reducing inter‐subgroup conflict or improving inter‐subgroup coordination within a common group is not the same as reducing interpersonal conflict. Subgroups nested within a common group are sensitive to maintaining their identity boundaries and will entrench themselves in inter‐subgroup conflict if those boundaries are seemingly being devalued, blurred, and ultimately lost within a common, superordinate identity (e.g., Crisp, Stone, & Hall, 2006; Dovidio, Gaertner, Pearson, & Lamoreaux, 2009; Giessner, Ullrich, & Van Dick, 2011). Resolving inter‐subgroup conflict, therefore, requires superordinate leaders to act as intergroup leaders in carefully navigating social categories and boundaries (Hogg, 2015; Hogg et al., 2012a). Uniting subgroups in such a way that inter‐subgroup hostility declines but subgroups do not feel that their distinctive identities are dismissed is a crucial task for an intergroup leader.

Promoting a collective identity while there is conflict between groups, therefore, becomes increasingly ineffective in improving intergroup relations and leaders should rely on a different approach. In promoting an overarching collective identity, which recategorizes all subgroup members into the superordinate group (Dovidio et al., 2009; Gaertner, Dovidio, Anastasio, Bachman, & Rust, 1993), leaders may only bolster intergroup tensions as group members resist the perceived threat to the distinctiveness of their subgroup identity (also see Rast et al., 2018; cf. Hornsey & Hogg, 2000b). Instead, the identity message promoted by the intergroup leader needs to focus on an *intergroup relational identity*, which recognizes and celebrates subgroup distinctiveness and defines subgroups explicitly in terms of their mutually promotive relationship with other subgroups within the common group to make the relationship an intrinsic part of their social identity (Hogg et al., 2012a; Rast, van Knippenberg, & Hogg, 2019).

Promoting an intergroup relational identity, relative to a collective identity, is the difference between advocating an understanding of group identity that sees the relationship with the other group as integral to that identity (e.g., what it means to be nurses is in part defined by the collaborative relationship between nurses, doctors, and hospital staff) and an understanding of identity that puts the emphasis on the shared superordinate group membership (e.g., nurses, hospital staff, and doctors are all health care providers or all part of this hospital). Intergroup relational identity rhetoric emphasizes that each group provides an important, distinct, and necessary function within the broader context of the shared group membership *and* that the functions of all subgroups are intertwined in that all subgroups are needed for the collaboration to be effective (e.g., doctors, nurses, and hospital staff all need to work together to provide effective health care). Focusing on the intrinsically collaborative and interdependent relationship between subgroups avoids entrenching subgroups in conflict and does not imply significant similarity among subgroups or dismiss subgroup social identities, therefore not inducing identity distinctiveness threat. Supporting these claims, group members who experienced a threat to the identity distinctiveness of their own group rated a leader more favourably when advocating an intergroup relational identity than an overarching collective identity, and this effect was reversed in the absence of identity distinctiveness threat (Rast et al., 2018). Similarly, promoting an intergroup relational identity enhances intergroup communication, resource sharing, and intergroup cooperation (Salem, Van Quaquebeke, Besiou, & Meyer, 2019; van der Stoep, Sleebos, van Knippenberg, & van de Bunt, 2020).

Viewing an intergroup leader as a trustworthy and non‐partisan source of identity information is a challenge of intergroup leadership. Trust, based upon being viewed as ‘one‐of‐us’, is an important basis for effective influence and leadership in groups (Barreto & Hogg, 2017). In intergroup leader situations, however, the leader can be viewed as being more closely affiliated with one subgroup than others. Indeed, the leader will often have clear roots in one of the subgroups (e.g., the leader's own political party, the leader's own functional area of expertise within the organization), which effectively casts them as a trusted ingroup member for one subgroup and a distrusted out‐subgroup member for the other subgroups (Duck & Fielding, 1999, 2003). For example, to Chemistry students an in‐subgroup leader would be affiliated with Chemistry, but a leader from Accounting would be an out‐subgroup leader. In‐subgroup and out‐subgroup leaders are specific to a context in which a superordinate group consists of two or more subgroups. Out‐subgroup intergroup leaders are in a precarious position because they must manage trust issues and avoid dismissing real subgroup differences, which could exacerbate the intergroup conflict they are trying to reduce (e.g., Rast et al., 2018; Riek, Mania, & Gaertner, 2006). Understanding the influence of the in‐subgroup versus out‐subgroup association, the role of subgroup affiliations, is important to development of intergroup leadership theory, intergroup relations, and the focus of the current studies.

### The current studies

We can expect that from an ingroup bias point of view, advocating an intergroup relational identity as compared with an overarching collective identity is more effective for out‐subgroup leaders than in‐subgroup leaders. Out‐subgroup leaders are expected to be biased towards the out‐subgroup (Duck & Fielding, 1999) and emphasizing collective identity may be understood to suggest an understanding of the collective identity that favours the out‐subgroup (Hogg et al., 2012a; Mummendey & Wenzel, 1999). This interpretation of out‐subgroup leadership is not suggested when the leader advocates an intergroup relational identity that underscores the distinctive identity of each subgroup as part of their collaborative, mutually defining relationship. Such a message from an out‐subgroup leader is therefore more likely to be embraced than a message emphasizing the collective identity. In‐subgroup leaders, in contrast, enjoy greater trust from the members of the in‐subgroup and thus will be less likely to raise fears of out‐subgroup favouritism when advocating an overarching collective identity. Indeed, advocating a collective identity message may even be understood to favour the leader's former group, the in‐subgroup, as per ingroup projection mechanisms (Mummendey & Wenzel, 1999). As a result, collective identity advocacy from an in‐subgroup leader may be as effective as, or more effective than (cf. Rast et al., 2018), advocacy of an intergroup relational identity for lessening ingroup bias.

Intergroup leadership is more challenging when people identify more strongly with their subgroup. With stronger subgroup identification, the distinctiveness of subgroup identity is more important to group members, which could increase conflict and even derogation between the subgroups (e.g., Jetten et al., 1997), and group members are more resistant to being subsumed in an overarching collective identity (van Leeuwen, van Knippenberg, & Ellemers, 2003). Therefore, how an intergroup leader manages the distinct subgroup identities will have a major impact on inter‐subgroup relations. As a result, as member identification with the subgroup is higher, leader advocacy of an intergroup relational identity will be more effective than leader advocacy of an overarching collective identity for lessening ingroup bias. Moreover, building on our analysis as outlined in the previous paragraphs, we can expect that this holds more so for out‐subgroup leaders than for in‐subgroup leaders, because the concerns with subgroup identity distinctiveness threat are stronger for out‐subgroup leaders.

**Figure 1:**
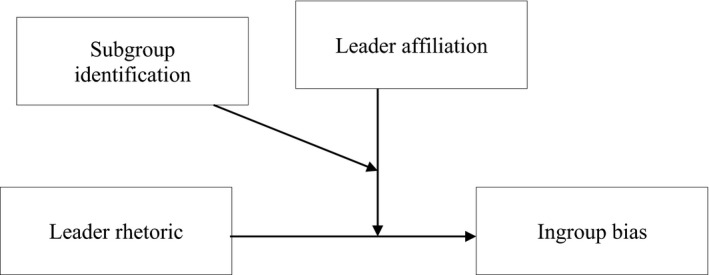
Visual representation of hypothesis for studies 1 and 2.

Thus, we suggest that ingroup bias will be influenced by the interaction of (1) subgroup identification, (2) whether the leader is an in‐subgroup or out‐subgroup member, and (3) whether the leader promotes an intergroup relational or collective identity, see Figure 1. More specifically, we predicted a three‐way interaction effect of these variables such that in mitigating ingroup bias, advocacy of an intergroup relational identity is more effective than advocacy of a superordinate collective identity for out‐subgroup leaders but not for in‐subgroup leaders, and more so with stronger subgroup identification. Predictions for stronger subgroup identification are the focus of these studies because social identity theory does not account for low subgroup identification (Hogg, 2018b).

We conducted two studies to test this three‐way interaction hypothesis. As stated previously, the two studies differed in the intergroup context and method employed because we wanted to test our hypotheses across different contexts and methods to go some way towards satisfying the contemporary call for replicability (e.g., Open Science Collaboration, 2015; Stangor & Lemay, 2016).

## Study 1

Study 1 was a quasi‐experiment investigating whether an out‐subgroup (vs. in‐subgroup) leader could improve intergroup relations by using rhetoric to promote either an intergroup relational or a collective identity. The study was conducted with psychology students at a university where the Psychology Department was housed within two faculties (Arts and Science), had students from both faculties, and was considering consolidating into either Arts or Science entirely. To induce an out‐subgroup student leader in this context, the leader would thus have to be from a department outside Arts and Science (we opted for a leader from Accounting, which is in the Business school). To induce an in‐subgroup leader at the same level of categorization (i.e., Psychology vs. Accounting), we framed the experimental context around the intended consolidation of Psychology within one faculty; foregoing the subdivision in Arts and Sciences, we introduced the in‐subgroup leader as from Psychology within the scenario that Psychology would unite within the faculty that was not the participants' faculty (i.e., Science for participants from Psychology‐Arts, Arts for participants from Psychology‐Science). The point here is to create a narrative that is constant across conditions while emphasizing Psychology as a united department rather than the Arts‐Science subdivision; the choice to focus on the scenario of Psychology being united in the other faculty than participants’ own faculty was arbitrary – the key point was that this allowed us to keep this constant.

To examine the hypotheses, we pretested department identification and then asked participants to read a topical vignette manipulating the rhetoric (intergroup relational vs. collective identity) from a supposed out‐subgroup or in‐subgroup leader. Participants subsequently evaluated both the in‐subgroup and out‐subgroup.

### Method

#### Participants and design

Participants were 178 undergraduate Arts or Science Psychology students (57.30% females, *n* = 102; 42.69% males, *n* = 76) at a large Canadian university.^1^ They ranged in age from 17 to 34 (*M* = 19, *SD* = 1.78) and were primarily first year undergraduate students (66%, *n* = 118) with some second year students (21.9%, *n* = 39) and the rest third and fourth years (11.7%, *n* = 21). The majority reported being Canadian (51.6%, *n* = 92), with the next largest nationality being Indian (29.7%, *n* = 53) and then Chinese (14.0%, *n* = 25). The study was described as a leadership study, and participants received partial course credit for their participation. There were two manipulated predictor variables, leader group membership (in‐subgroup, out‐subgroup) and leader rhetoric (collective identity, intergroup relational identity), one measured predictor variable (ingroup identification), and one main dependent measure (ingroup bias).

#### Procedure and measures

Participants were recruited through the Psychology Department subject pool to complete a study on the 'psychology of leadership'. Participants entered a laboratory, sat in separated cubicles at computer workstations, and completed the study via Qualtrics, an online study platform. Participants began by reporting basic demographic information (age, gender, year in school, nationality, and their faculty) and the strength of their identification with the Psychology Department via four statements, adapted from previous social identity research (e.g., Grant, Hogg, & Crano, 2015). The statements focused on (1) how important to their identity the department was, (2) how frequently they thought about themselves as a member of the department, (3) to what extent the department influenced their life choices, and (4) to what extent the department influenced their daily decisions; 1 = *strongly disagree*, 9 = *strongly agree* (higher scores indicate stronger identification; *α* = .89).

Then participants were told that the Psychology Department was considering moving out of the student's home faculty, either the Arts or Science as reported in the demographics, and into their non‐home faculty. For example, those who reported Faculty of Arts‐Psychology were told the Psychology Department was considering a move into the Faculty of Science. In addition, participants were told a student leader representing students from all departments would assist during this transition and the participant would read a brief statement from the prospective student leader. The in‐subgroup or out‐subgroup manipulation was contained within the instructions for the vignette, in which the participants were told the student was from Psychology (in‐subgroup) or Accounting (out‐subgroup), and the vignette delivered the leader rhetoric manipulation (modified from Rast et al., 2018). Specific manipulations for intergroup relational identity rhetoric in which the leader promoted ‘…maintaining their distinct and separate group identities to achieve common goals’, whereas specific manipulations for collective identity rhetoric in which the leader promoted ‘…must understand they are similar to one another and work together to achieve common goals’.

After reading the vignette, participants reported their ingroup bias. Adapted from previous intergroup relations research (Wright, Aron, McLaughlin‐Volpe, & Ropp, 1997), participants rated their own faculty (Arts or Science) and also the out‐subgroup faculty on six semantic differentials: ‘[… faculty] students are…’ (1) cold/warm, (2) negative/positive, (3) hostile/friendly, (4) suspicious/trusting (e.g., 1 = *cold*, 9 = *warm*) and ‘[… faculty] students deserve…’ (5) contempt/respect, and (6) disgust/admiration (e.g., 1 = *contempt*, 9 = *respect*).

The six semantic differentials formed a reliable scale for both in‐subgroup and out‐subgroup evaluations (α = .85 for in‐subgroup attitude and α = .83 for out‐subgroup attitude), with higher scores signifying a more positive attitude towards the specific faculty. Following procedures used by Hornsey and Hogg (2000b) and Rast et al. (2018), out‐subgroup attitude was subtracted from in‐subgroup attitude to create a measure of ingroup bias. Positive scores indicate more positive evaluation of the in‐subgroup than the out‐subgroup, and negative scores vice versa.

Finally, participants were thanked for their time and fully debriefed.

### Results

Table 1 displays alpha reliabilities, means, standard deviations, and intercorrelations of measured variables. There were two manipulated, predictor variables (leader group membership, leader rhetoric), one measured predictor variable (ingroup identification), and one main dependent measure (ingroup bias). Because one predictor was continuous, the data were analysed using hierarchical multiple regression. Following Aiken and West (1991), predictor variables were centred, interaction terms calculated, and simple slopes analyses conducted for significant interactions.

**Table 1:** Study 1: Reliabilities, means, *SD*s, and intercorrelations of all key variables

Variable	α	*M*	*SD*	2	3	4	5	6
1. Leader rhetoric	–	1.50	0.50	.02	−.10	−.05	−.09	.04
2. Group membership	–	1.51	0.50	–	−.06	.08	.05	.04
3. Identification with ingroup	.89	3.65	1.42	–	–	.21	.20	.03
4. In‐subgroup attitude	.85	6.78	1.27	–	–	–	.57	.52
5. Out‐subgroup attitude	.83	6.78	1.19	–	–	–	–	−.41
6. Ingroup bias	–	−0.01	1.15	–	–	–	–	–

Note.

Means (*N* = 178) for identification with ingroup can take values between 1 and 7 and means for in‐subgroup and out‐subgroup attitudes can take values between 1 and 9, with 7 or 9 indicating more of the property described except leader rhetoric and group membership, which are binary (1, 2) variables and ingroup bias, which is (in‐subgroup attitude – out‐subgroup attitude).

#### Background variables

Regressing the three predictor variables and their interactions onto participant age, year in school, nationality, and gender revealed a significant effect of identification on gender and of leader rhetoric on year in school. Controlling for gender and year in school in subsequent analyses, however, did not change the pattern of results. Therefore, the reported analyses reflect our original design.

#### Ingroup bias

The hierarchical linear regression of leader rhetoric, ingroup identification, and leader group membership on ingroup bias was not statistically significant at Step 1, adj*R* ^2^ = −.01, *F*(3, 173) = 0.24, *p* = .87, nor did any of the main effects approach statistical significance. The inclusion of the two‐way interactions at Step 2 was statistically significant, Δ*R* ^2^ = .07, *F*(3, 170) = 3.80, *p* = .01. Only the two‐way interaction between leader group membership and ingroup identification was statistically significant, β = 0.23, *t*(173) = 3.02, *p* = .003, 95% CI = [0.08, 0.38]. These effects, however, were qualified by the predicted and statistically significant three‐way interaction included at Step 3, Δ*R* ^2^ = .02, *F*(1, 169) = 4.36, *p* = .03. As hypothesized, there was a three‐way interaction between leader group membership, leader rhetoric, and ingroup identification, β = 0.16, *t*(169) = 2.09, *p* = .03, 95% CI = [0.01, 0.30].

**Figure 2:**
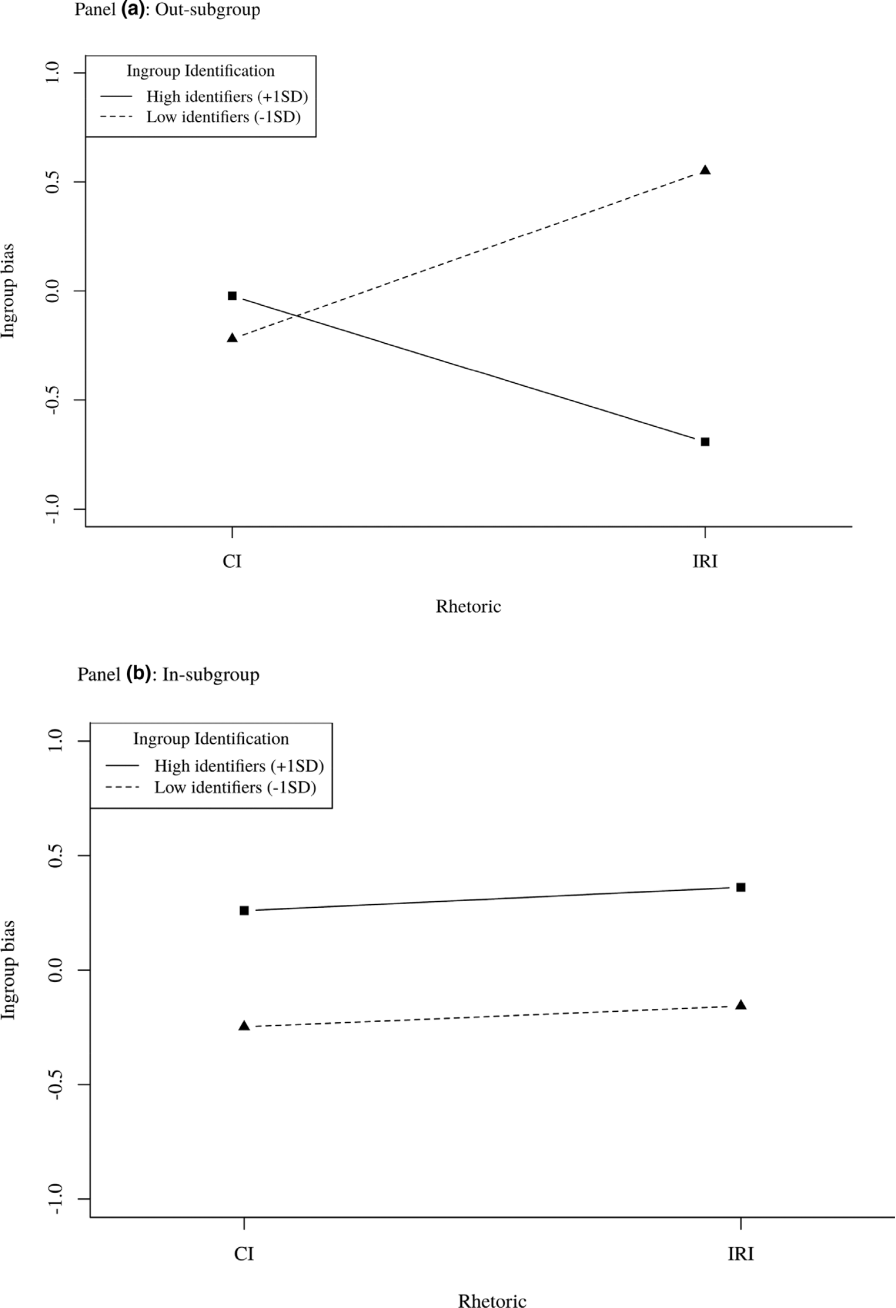
Study 1: Ingroup bias across levels of leader rhetoric and ingroup identification.

As shown in Figure 2, simple slopes revealed weakly identified participants with an out‐subgroup leader reported greater ingroup bias when the leader promoted an intergroup relational than collective identity, *β* = .33, *t*(169) = 2.12, *p* = .04, 95% CI = [0.02, 0.64], whereas strongly identified participants reported less ingroup bias when the out‐subgroup leader promoted an intergroup relational than collective identity, *β* = −.29, *t*(169) = −1.94, *p* = .054, 95% CI = [−0.59, 0.00]. When the leader promoted an intergroup relational identity, changing the leader's group membership (from out‐subgroup to in‐subgroup) was associated with less ingroup bias among weakly identified participants, *β* = −.31, *t*(169) = −2.18, *p* = .03, 95% CI = [−0.58, −0.03], but increased ingroup bias among highly identified participants, *β* = .46, *t*(169) = 2.96, *p* = .004, 95% CI = [0.15, 0.77]. Finally, an out‐subgroup leader promoting an intergroup relational identity was associated with less ingroup bias as participants identified more strongly with their subgroup, *β* = −.54, *t*(169) = −3.30, *p* < .001, 95% CI = [−0.86, −0.22], whereas an in‐subgroup leader promoting an intergroup relational identity marginally strengthened ingroup bias, *β* = .22, *t*(169) = 2.10, *p* = .09, 95% CI = [−0.03, 0.48]. No other simple slopes were statistically significant.

### Discussion

Study 1 examined whether an out‐subgroup (vs. in‐subgroup) leader could reduce ingroup bias and thus improve intergroup relations, through promoting an intergroup relational compared to collective identity rhetoric. The pattern of results generally supported our hypothesis. An out‐subgroup leader emphasizing an intergroup relational identity was associated with less ingroup bias among high subgroup identifiers, whereas promoting a superordinate collective identity was associated with lower ingroup bias among low identifiers. For an in‐subgroup leader, in contrast, whether the leader advocated an intergroup relational identity or a collective identity was inconsequential for ingroup bias. Given the large number of departments in a university and the complexity of a student leader representing students in those departments and influencing outcomes for each department, we sought to replicate these findings in a common situation outside the university context.

## Study 2

In Study 1, participants read information indicating their nested group context would change and how a potential leader would assist that change and then reported their intergroup bias. A second study was conducted to test the same hypotheses as Study 1, but in a different intergroup context and with a different method. Not only is it important to demonstrate consistency in outcomes across different contexts (Stangor & Lemay, 2016), but it is also important to further clarify the intergroup context being studied. Study 1 allowed us to test our hypotheses with participants immersed in the intergroup context. Study 2 allowed us to enhance mundane realism, while maintaining the experimental nature of Study 1 by implementing a scenario methodology. Like Study 1, Study 2 was a quasi‐experiment in which leader group membership and leader rhetoric were manipulated and participant ingroup identification was measured. The key intergroup difference was that we moved from an intergroup context within a university to one between rival cities within a shared Canadian province. By testing our hypotheses from Study 1 in a different methodological and intergroup context, we sought to demonstrate the robustness of our findings.

Similar to Study 1, we sought to use a backdrop that would enhance the psychological realism of Study 2. Study 2 took place during a provincial election cycle. Participants were citizens of one of the two major cities in the province, and the leader was ostensibly from one of the two major cities. The superordinate group was the participants' province and the subgroups were the participants' current city of residence and a second major city in the province with which there is a contentious relationship. There is often perceived competition between the two cities and how each city is represented within the province.

### Method

#### Participants and design

Participants were 243 undergraduate psychology students (64.19% females, *n* = 156; 35.80% males, *n* = 87) at a large Canadian university.^2^ They ranged in age from 18 to 30 (*M* = 19, *SD* = 2.30) and were primarily first year undergraduate students (53.9%, *n* = 131) and second year undergraduate students (28.8%, *n* = 70), while the remaining participants were either in or beyond their third year (17.2%, *n* = 42). The majority reported being Canadian (51.4%, *n* = 125), with the next largest nationality being Indian (12.7%, *n* = 31) and Chinese (10.6%, *n* = 26). A majority of the remaining participants reported being from either southeast Asian (12.7%, *n* = 31), Middle Eastern (3.2%, *n* = 8), and African (2.8%, *n* = 7) regions. The study was described as a leadership study, and participants received partial course credit for their participation. There were two manipulated, predictor variables leader group membership (in‐subgroup, out‐subgroup) and leader rhetoric (collective identity, intergroup relational identity), one measured predictor variable (ingroup identification), and one dependent measure (ingroup bias).

#### Procedure and measures

Participants were recruited through the Psychology Department subject pool to complete a study on the 'psychology of leadership'. Participants entered a laboratory, sat in separated cubicles at computer workstations, and completed the study via Qualtrics, an online study platform. Participants started by reporting how often they identified as a resident of their city via two statements, *r*(240) = .73, *p* < .001, adapted from previous social identity research (e.g., Grant et al., 2015), measuring strength of identification with their city of residence. The two questions were, ‘How often are you aware of being an [city of residence]’, and ‘How often do you think about your identity as being an [city of residence]?’ (1 = *Never*, 5 = *Always*). Higher scores indicated stronger identification.

Participants were then told they would read a hypothetical news article and answer questions afterwards. The in‐subgroup or out‐subgroup manipulation was in the hypothetical news article which indicated the potential political leader was either from the participants' current city of residence or from a nearby city. The hypothetical news article also delivered the leader rhetoric manipulation and was a slightly modified version of the leader rhetoric manipulation from Study 1.

After reading the vignette, participants completed a manipulation check and reported their ingroup bias, using the same measure from Study 1 (α = .90 for in‐subgroup attitude; α = .91 for out‐subgroup attitude). Following the same procedure as Study 1, out‐subgroup attitude was subtracted from in‐subgroup attitude to create a measure of ingroup bias.

Then, participants provided basic demographic information (age, gender, year in school, nationality) and their political orientation. Finally, participants were thanked for their time and fully debriefed.

### Results

Table 2 displays alpha reliabilities, means, standard deviations, and intercorrelations of measured variables. There were two manipulated, predictor variables (leader group membership, leader rhetoric), one measured predictor variable (ingroup identification), and one dependent measure (ingroup bias). Because one predictor was continuous, the data were analysed using hierarchical multiple regression. Following Aiken and West (1991), predictor variables were centred, interaction terms calculated, and simple slopes analyses conducted for significant interactions.

**Table 2:** Study 2: Reliabilities, means, *SD*s, and intercorrelations of all variables

Variable	α	*M*	*SD*	2	3	4	5	6	7
1. Leader rhetoric	–	1.49	0.50	.00	−.03	.02	.01	.02	.05
2. Group membership	–	1.49	0.50	–	.05	.02	−.00	.02	.03
3. Identification with ingroup	–	2.69	0.91	–	–	.26	.01	.29	.20
4. In‐subgroup attitude	.89	7.39	1.37	–	–	–	.61	.51	.04
5. Out‐subgroup attitude	.91	6.61	1.27	–	–	–	–	−.37	.02
6. Ingroup bias	–	0.78	1.17	–	–	–	–	–	–

Note.

Means (*N* = 223) for identification with ingroup can take values between 1 and 5 and means for leader evaluation and in‐subgroup and out‐subgroup attitudes can take values between 1 and 9, with 5 or 9 indicating more of the property described except leader rhetoric and group membership, which are binary (1, 2) variables and ingroup bias, which is (in‐subgroup attitude – out‐subgroup attitude).

#### Background variables and manipulation check

Regressing the three predictor variables and their interactions onto participant year in school, age, nationality, political orientation, and gender did not reveal any significant effects (*p*s > .11). Therefore, these variables were excluded from all subsequent analyses.

As a manipulation check, participants were asked whether the potential political leader was from the participant's resident city or a nearby city. Whether the participant correctly identified from which city the leader originated served as our manipulation check of leader group membership. Participants who did not correctly identify the leader's group membership were excluded from data analysis, bringing the sample to 223.

#### Ingroup bias

The hierarchical linear regression of leader rhetoric, ingroup identification, and leader group membership on ingroup bias was statistically significant at Step 1, adj*R* ^2^ = .08, *F*(3, 219) = 7.00, *p* < .001. A main effect for ingroup identification emerged such that stronger identification was associated with more ingroup favouritism, β = 0.30, *t*(219) = 4.56, *p* < .001, 95% CI = [0.17, 0.42]. The inclusion of the two‐way interactions at Step 2 did not account for a statistically significant amount of variance, Δ*R* ^2^ = .01, *F*(3, 216) = 0.91, *p* = .43, nor did any of the two‐way interactions approach statistical significance. These effects, however, were qualified by the hypothesized three‐way interaction including at Step 3, Δ*R* ^2^ = .02, *F*(1, 215) = 3.69, *p* = .05. There was a three‐way interaction between leader group membership, rhetoric, and ingroup identification, β = 0.12, *t*(215) = 1.92, *p* = .05, 95% CI = [0.00, 0.25].

**Figure 3:**
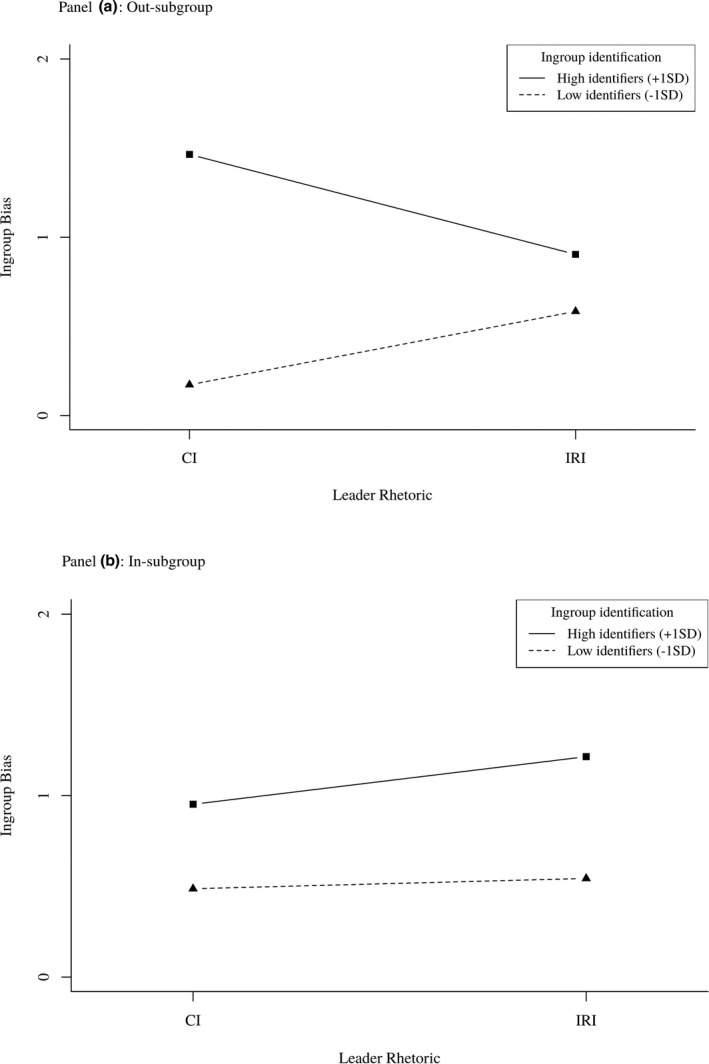
Study 2: Ingroup bias across levels of leader rhetoric and ingroup identification.

As shown in Figure 3, simple slopes revealed strongly identified participants reported marginally less ingroup bias when the out‐subgroup leader promoted an intergroup relational than collective identity, *β* = −.24, *t*(215) = −1.77, *p* = .08, 95% CI = [−0.50, 0.03]. An out‐subgroup leader promoting a collective identity was associated with more ingroup bias as participants identified more strongly with their group, *β* = .55, *t*(215) = 4.33, *p* < .001, 95% CI = [0.30, 0.80], as did an in‐subgroup leader promoting an intergroup relational identity, *β* = .29, *t*(215) = 2.22, *p* = .03, 95% CI = [0.03, 0.54]. No other simple slopes approached statistically significance.

### Discussion

Study 2 by and large replicated the results obtained in Study 1. For out‐subgroup leaders there was a crossover pattern such that advocating an intergroup relational identity was associated with lower ingroup bias than advocating a collective identity for high identifiers but with higher ingroup bias for low identifiers. For in‐subgroup leaders, leader advocacy was overall less influential for ingroup bias. Replicating the core findings across both studies bolsters our confidence in the conclusions based on these findings.

## Meta‐analytic summary

We conducted a meta‐analytic summary of our two studies and reported findings from a larger aggregate sample of 401 participants. Following the recommendations of Goh, Hall, and Rosenthal (2016), we used fixed effects in which the mean effect size (i.e., mean correlation) was weighted by sample size. All correlations were then Fishers’s *Z* transformed for analyses and converted back to Pearson's correlations for presentation. We first converted our *t*‐values into Pearson's correlation for ease of analysis. Overall, the effect for an out‐subgroup leader and strongly identified participants was significant (*M_r_* = .13, *Z* = 2.66, *p* < .01, two‐tailed), such that an out‐subgroup leader promoting an intergroup relational compared to a collective identity reduced ingroup bias. In addition, the effect for an out‐subgroup leader and weakly identified participants was significant (*M_r_* = .14, *Z* = 2.75, *p* < .01, two‐tailed), such that an out‐subgroup leader promoting an intergroup relational compared to a collective identity increased ingroup bias.

## General discussion

Although social psychologists have long studied ways in which to reduce ingroup bias, discrimination, and intergroup conflict (see Dovidio & Gaertner, 2010; Yzerbyt & Demoulin, 2010), the focus has rarely if ever been explicitly on the role played by a leader. In this article, we have adopted a perspective in which intergroup conflict, and associated ingroup bias, is typically inter‐subgroup conflict nested within a superordinate identity. In this situation, the leader of the superordinate group, and therefore intergroup leadership, plays a critical role in mending, or exacerbating, subgroup conflict.

Framed by social identity theory (for overview see Hogg, 2018b) and research on reducing conflict between groups (e.g., Gaertner & Dovidio, 2000; Hornsey & Hogg, 2000a), we grounded our research in intergroup leadership theory (Hogg, 2015; Hogg et al., 2012a). Intergroup leaders who are trying to mend hostile relations between subgroups must be very careful to not promote a collective identity that threatens the distinctiveness and associated cherished identities of subgroups. Instead, they need to promote an intergroup relational identity that celebrates subgroup distinctiveness and defines the social identity of the subgroups in terms of the cooperative and interdependent relationship between them. This is a challenge that is accentuated when (1) subgroup members identify strongly with their subgroups and when (2) the superordinate leader is considered not ‘one of us’ because they are viewed as being an out‐subgroup member.

From this analysis, we hypothesized that (1) strength of subgroup identification would interact with (2) the superordinate leader's subgroup membership and (3) whether they delivered an intergroup relational or collective identity message to influence ingroup bias. We conducted two studies with different intergroup and methodological contexts: Study 1 emphasized faculty affiliations within a university department, and Study 2 emphasized rival cities within a Canadian province using a scenario method. We sought to test our hypotheses in different intergroup and methodological contexts to address generalizability and the contemporary call for replicability (e.g., Open Science Collaboration, 2015; Stangor & Lemay, 2016).

The pattern of results across the two studies, and confirmed with a meta‐analysis, supports our predictions. We found that an out‐subgroup leader promoting an intergroup relational identity as compared with a collective identity invited lower ingroup bias among strongly identifying group members and invited higher ingroup bias among weakly identifying group members, whereas ingroup bias was not affected by identity advocacy when the leader was associated with the in‐subgroup. Although the pattern of results across the studies have some differences, such as lingering ingroup bias from high identifiers in the second study, participants being told the study was hypothetical could have made the change in ingroup bias less drastic. Hypothetical rhetoric, however, was still effective in reducing ingroup bias among high identifiers. Our theory and findings extend intergroup leadership theory by speaking to the role of the in‐subgroup or out‐subgroup affiliation of the intergroup leader and of member identification with their own subgroup and does so in a way that is well‐aligned with intergroup leadership theory and findings (Hogg et al., 2012a; Rast et al., 2018). These results speak to the importance of identity and leadership in intergroup relations and lend additional support to intergroup leadership theory and the importance of understanding intergroup leadership situations. Future research can expand on the results of our meta‐analysis to include additional moderators for the effect of an intergroup leader for improving ingroup bias.

### Limitations and future directions

Studies 1 and 2 provided support for the theoretical framework and hypotheses under investigation, although there are some important considerations. The first potential limitation is the lack of a direct manipulation check for leader rhetoric in both studies. Although it is good practice to employ manipulation checks, we sought to keep these studies as short as possible. In addition, the leader rhetoric manipulations were modified from existing published research (Rast et al., 2018), in which a pilot study testing the effectiveness of this manipulation on a similar sample was conducted. Similarly, the main dependent measure was also modified from this earlier work, further demonstrating the effectiveness of the leader rhetoric manipulation onto the ingroup bias measure.

Another potential limitation is the lack of an identity distinctiveness threat measure. Our theory implies that individuals facing an out‐subgroup leader and identifying more strongly with their group would be more likely to interpret the situation as a distinctiveness threat (i.e., and therefore respond more positively to intergroup relational identity rhetoric than to collective identity rhetoric), and our findings are consistent with this reasoning as well as with previous research on intergroup leadership and identity distinctiveness threat (Rast et al., 2018). Even so, it must be noted that we do not have evidence of an experienced identity distinctiveness threat that would make our interpretation of these findings stronger.

A constraint on the generalizability of our findings relates to the samples used in the two studies presented. Despite replicating the key findings across two studies, Studies 1 and 2 were both conducted with an undergraduate Psychology sample at a large, public Canadian university because they were convenient samples. However, social identity research, particularly social identity research on leadership, is quite robust with successful replications conducted by different research groups on six continents using different measures and operationalizations, including archival (secondary) data, field studies, and laboratory studies (see van Knippenberg, 2011). Therefore, we believe the results of these studies will be reproducible across student and non‐student samples using different operationalizations and context.

### Conclusion

It is important to understand how effective intergroup leaders can successfully navigate social identity processes to improve intergroup relations and enhance intergroup cooperation. Traditional intergroup conflict theories and strategies do not account for this all too common inter‐subgroup conflict within an intergroup leadership context. That context is prime for conflict escalation due to reactions towards dismissing real subgroup differences and the issue of the loyalties of an out‐subgroup leader. Out‐subgroup leaders are seen as 'one of them' rather than 'one of us'. For instance, US President Donald Trump is perceived as 'one of us' with the Republican‐controlled Senate, but he is perceived as 'one of them' by the Democrat‐controlled House of Representatives. Resolving potential conflict among the different sides of Congress, therefore, would require a strategy that considers how the groups perceive him while he resolves conflict. On a smaller scale, effective intergroup leadership could be effective applied in businesses, particularly considering how frequently departments need to work with each other (e.g., van der Stoep et al., 2020) or joint ventures between businesses (Hambrick, Li, Xin, & Tsui, 2001).

Intergroup leadership theory proposes that out‐subgroup leaders will be more effective if they foster an intergroup relational identity among strongly identified group members (Hogg et al., 2012a). Across two studies, we provided evidence to support this hypothesis: endorsing an intergroup relational identity effectively lowered ingroup bias for otherwise ardent defenders of the ingroup. Evoking an intergroup relational identity was effective for out‐subgroup leaders when members identified strongly with their group. An out‐subgroup leader in an intergroup context is an inevitable situation and understanding how inter‐subgroup relations can be improved with such a leader would increase our understanding of intergroup conflict, bias, and discrimination.

## Conflicts of interest

All authors declare no conflict of interest.

## Author contributions

Christine Kershaw (Conceptualization; Data curation; Formal analysis; Investigation; Methodology; Project administration; Resources; Software; Validation; Visualization; Writing – original draft; Writing – review & editing) David E. Rast (Conceptualization; Data curation; Formal analysis; Funding acquisition; Methodology; Project administration; Supervision; Validation; Writing – review & editing) Michael A. Hogg (Conceptualization; Writing – review & editing) Daan van Knippenberg (Conceptualization; Writing – review & editing).

## Footnotes

^1^
Following recent recommendations (Aberson, 2019), we conducted a priori and post‐hoc power analyses using R (R Core Team, 2019). A priori power analysis, in which we estimated parameter estimates and then resampled this model 10,000 times, yielded a sample size recommendation of 185 for .95 power and *p* = .05. Post‐hoc power analyses, using the obtained model parameters, demonstrated our statistical models were adequately powered for ingroup bias (power = .86).

^2^
Following the same procedure as Study 1, a priori power analysis was conducted for Study 2. We estimated parameter estimates and then resampled this model 10,000 times, yielded a sample size recommendation of 216 for .95 power and *p* = .05. Post‐hoc power analyses, using the obtained model parameters, demonstrated our statistical models were adequately powered for ingroup bias (power = .87).
